# Queer in Chem: Q&A with Dr Joshua Makepeace

**DOI:** 10.1038/s42004-023-00967-6

**Published:** 2023-09-30

## Abstract

Dr Josh Makepeace is an Associate Professor in Materials Chemistry and UK Research and Innovation (UKRI) Future Leaders Fellow at the University of Birmingham. Raised in Australia, Josh started his journey in chemistry research at Flinders University of South Australia, investigating the origin of hematite crystals in William Bligh’s naval logbooks, and the detection of pesticides in waterways.

**Figure:**
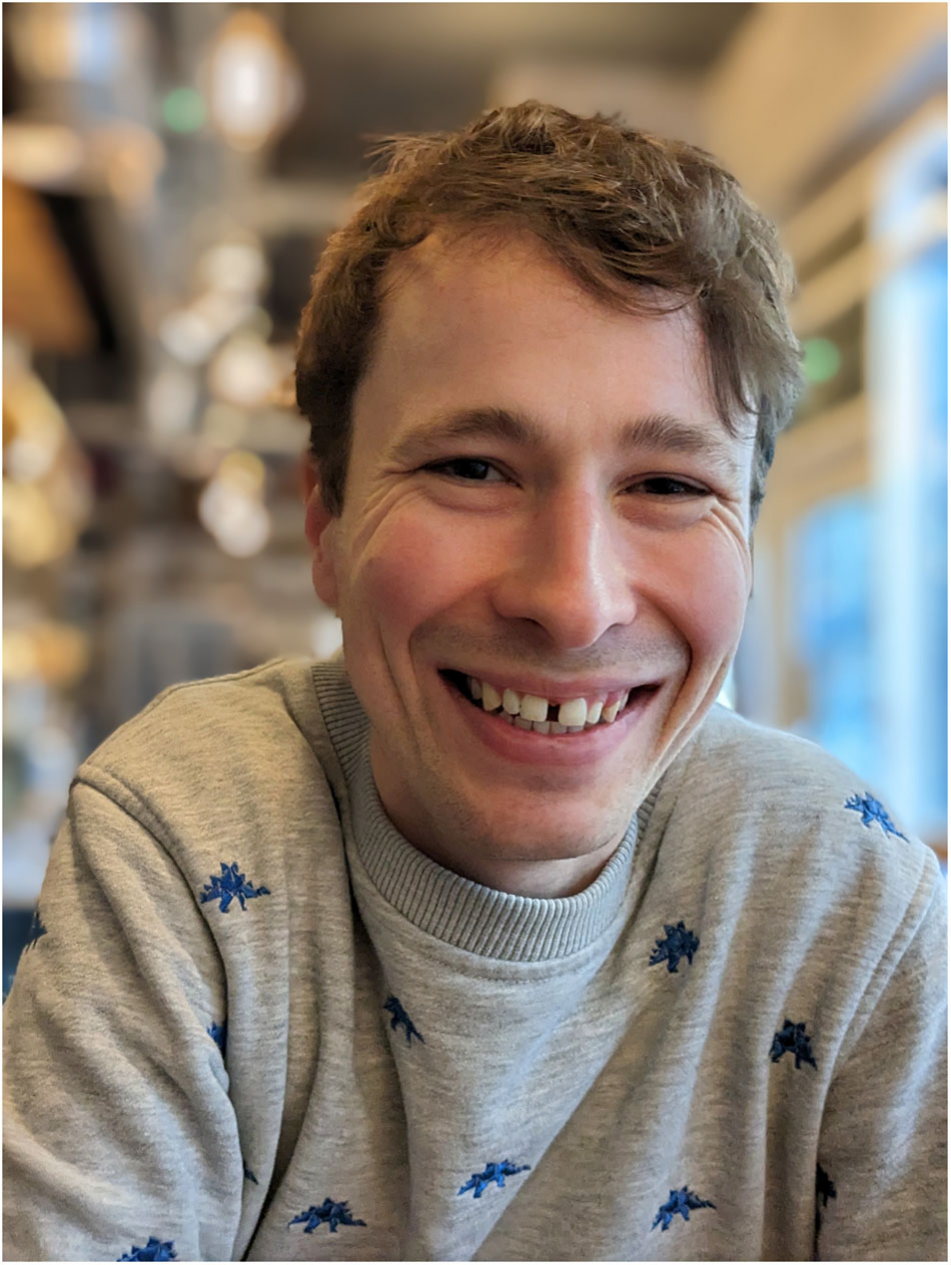
Joshua Makepeace

Josh moved to the UK in 2010 to pursue his doctoral studies on hydrogen storage materials at the University of Oxford. Following this, he worked at the ISIS Neutron and Muon Source and as a Junior Research Fellow at St John’s College, Oxford, before starting in Birmingham in late 2019. Josh leads a research group which is exploring the development of new catalysts and materials for energy storage applications, with a particular focus on green ammonia as a sustainable fuel. Josh is the 2023 recipient of the Royal Society of Chemistry’s Environment, Sustainability and Energy Early Career Prize.

Why did you choose to be a scientist?

I’d always enjoyed the excitement of finding out more about how the world works, and particularly the experimental side of chemistry; it never stops being a thrill to see new materials being made in the lab. However, what pushed me towards making science a career was the opportunity to use my understanding to help address environmental challenges such as climate change and pollution.

What scientific development are you currently most excited about?

It’s too difficult to list just one! It’s really exciting to see the creativity that’s been unleashed in the scientific community in response to the challenge of transitioning to a sustainable energy system. There’s a whole host of exciting developments, from new photovoltaics and batteries to catalysts for sustainable fuel transformations. I love seeing these new technologies at the beginning of their journey and thinking about how our world might be transformed by them.

What direction do you think your research field should go in?

In energy materials research, I think it’s important that we maintain research that’s focused on different timescales and levels of application. It’s crucial that we’re in there now helping design materials which will rapidly address today’s challenges. However, we also need to continue being creative and dreaming big about the possibilities for future devices that seem far-fetched today.

Why do you think it is important to feel comfortable enough to bring your whole self to work?

If you feel like you’re having to censor aspects of your identity at work, it leads to a kind of tension; that means it’s difficult to fully engage with your work-life. Finding that feeling of belonging at work helps you do your job more confidently and makes you feel more part of a community. For me, that makes me better at my job.

Have you faced any challenging situations in your professional life as a result of your queer or trans identity? What did you or others learn from these experiences?

Early on in my research career I struggled with that sense of belonging, as I didn’t know any LGBTQ+ chemists, which made it hard to see a future there for myself. That’s gotten easier as I’ve met more and more role models, and I think I’ve had quite a privileged experience to be honest. I’m a cis white man and knowing how I struggled with belonging makes me keenly aware of the far greater barriers which other parts of the LGBTQ+ community are currently facing.

How can individual scientists support and celebrate their LGBTQ+ colleagues?

When I started my current job, the fact that many colleagues were sensitive in their use of language around gender and sexuality gave me space to define myself on my own terms. Those things are very powerful: if you feel like you’re having to push back against assumptions right from the beginning, it can make things much less comfortable. It’s also incredibly heartening when people who aren’t LGBTQ+ are prepared to make their voice heard in defense of their colleagues. Ultimately an accepting and diverse workplace is better for everyone.

What action(s) do you feel employers in chemical research should take to make a difference for LGBTQ+ scientists?

As a university employee, I think it’s crucial that our employers recognise that they have significant international influence, through the student and researcher communities they foster through to the global reach of the research that’s done. Given that, it’s so important that universities realise that taking an active stance in support of LGBTQ+ scientists ripples out to places that have far more hostile environments for LGBTQ+ people. This is also true for chemical research companies more broadly, which often have international businesses or supply chains.

*This interview was conducted by the editors of Communications Chemistry*.

